# Low High-Density Lipoprotein Cholesterol Predisposes to Coronary Artery Ectasia

**DOI:** 10.3390/biomedicines7040079

**Published:** 2019-10-07

**Authors:** Jamal Jafari, Aner Daum, Jihad Abu Hamed, Azriel Osherov, Yan Orlov, Chaim Yosefy, Enrique Gallego-Colon

**Affiliations:** Cardiology Department, Barzilai University Medical Center, Hahistadrout St 2, Ashkelon 30604, Israel; jafari@barzi.health.gov.il (J.J.); aner.daum@gmail.com (A.D.); jihada@bmc.gov.il (J.A.H.); uziosherov@yahoo.com (A.O.); iano@bmc.gov.il (Y.O.); chaimy@bmc.gov.il (C.Y.)

**Keywords:** coronary artery ectasia, coronary artery aneurysm, high-density lipoprotein cholesterol, low-density lipoprotein cholesterol, hemoglobin, coronary artery disease, atherosclerosis

## Abstract

Coronary Artery Ectasia (CAE) is a phenomenon characterized by locally or diffuse coronary artery dilation of one or more coronary arteries. In the present study, the prevalence of acquired coronary ectasia and coronary risk factors for CAE was analyzed in patients undergoing cardiac catheterization for suspected ischemic heart disease. We retrospectively analyzed 4000 patients undergoing coronary angiography for suspected coronary artery disease at our cardiac catheterization unit, and a total of 171 patients were selected. The study group was divided into three groups, 65 patients with CAE, 62 patients with significant obstructive coronary artery disease, and 44 patients with normal coronary angiograms as a control group. A negative correlation was observed between high-density lipoprotein cholesterol (HDL-C) and the presence of CAE (r = −0.274, *p* < 0.001). In addition, HDL-C (OR, 0.858; CI, 0.749–0.984; *p* = 0.029), low-density lipoprotein cholesterol (LDL-C)/HDL-C ratio (OR, 1.987; CI, 1.542–2.882; *p* = 0.034), and hemoglobin (OR, 2.060; CI, 1.114–3.809; *p* = 0.021) were identified as independent risk factors for the development of CAE. In fact, we observed that a one-unit increase in HDL-C corresponded to a 15% risk reduction in CAE development and that each unit increase in hemoglobin could potentially increase the CAE risk by 2-fold. Low HDL-C could significantly increase the risk of developing CAE in healthy individuals. Elevated hemoglobin could predispose to subsequent dilation and aneurysm of the coronary artery. This work suggests that disordered lipoprotein metabolism or altered hemoglobin values can predispose patients to aneurysmal coronary artery disease.

## 1. Introduction

Coronary Artery Ectasia (CAE) is defined as local or diffuse coronary artery dilation of more than 1.5 times the diameter of the adjacent segment affecting one or more coronary arteries [1]. The prevalence of CAE in angiographic series varies between 1.4% and 10% [1,2,3]. Several factors have been associated with CAE, including lipoprotein concentration, inflammatory milieu, renin-angiotensin system, homocysteine levels, insulin, nitric oxide, and atherosclerosis [4]. Aneurysmal dilation in CAE is often associated with increased blood viscosity and turbulent flow, potentially leading to acute coronary artery infarction without total occlusion [5,6]. The coronary artery surgery study (CASS) showed that upon CAE diagnosis, the five-year mortality rate in patients with the coronary disease was 25–29.1% [1,2,3,4,5,6,7]. In CAE patients, the thrombogenic potential of ectatic arteries represents a clinical complication for interventional cardiologists despite the efficacy of anticoagulation therapy. Indirect pieces of evidence in cases of familial hypercholesterolemia have suggested an interesting association between plasma lipoproteins and CAE progression. Research by Sudhir and collaborators showed that lower high-density lipoprotein cholesterol (HDL-C) (*p* = 0.003) and, to a lesser extent, higher low-density lipoprotein cholesterol (LDL-C) (*p* = 0.07) were strongly associated with CAE progression in heterozygous familial hypercholesterolemia [8]. In the present study, we examined the prevalence of acquired coronary ectasia and coronary risk factors for CAE in patients undergoing cardiac catheterization.

## 2. Experimental Section

### 2.1. Study Population

For this retrospective cohort study, we analyzed detailed patient data collected in the cardiac catheterization unit from 2014 until 2018. Based on clinical indications, coronary angiography was performed to investigate ischemic heart diseases. The study analyzed 4000 patients undergoing catheterization at our unit, with a total of 171 patients selected for the study. The patients were matched by age, gender (male), and catheterization time. The population study was divided into three groups, ectatic (CAE), obstructive with coronary artery disease (CAD), and the control group with normal coronary angiograms. The study protocol adhered to the Declaration of Helsinki and was approved by the institutional review board of Barzilai Medical Center (BRZ-0005-14, approval date 02/02/2014, and further extended on 27/02/2016), Ben-Gurion University, Israel. Written informed consent was obtained from the patients to participate in the study.

### 2.2. Data Collection

In patients undergoing coronary artery angiography, CAE was diagnosed if at least one coronary artery was 1.5-fold or greater than the adjacent normal coronary artery segment [1]. In addition, coronary artery dilation, not accompanied by significant coronary artery stenosis, was incorporated in the CAE group [9]. Patients with stenosis of more than 50% of the diameter of the artery and no CAE were included in the obstructive group. In this study, the exclusion criteria included (a) patients under 18 years of age, (b) coronary artery bypass grafting, extensive coronary artery stenosis, malignancy, liver, kidney, acute or chronic inflammatory disease, and (c) patients not matched by age, gender (male), and catheterization time. For all routine echocardiography exams, iE33 echo machine (Philips Medical Systems, Andover, MA, USA) was used. All images were digitally stored for offline analysis (QLAB 10.0 cardiac 3DQ, Philips Medical Systems, Andover, MA, USA). For comorbidities, the following cut-off values were considered: blood pressure of >140/90 mm Hg in repeated measurements for arterial hypertension, fasting plasma glucose levels ≥126 mg/dL for diabetes mellitus, a total serum cholesterol level >200 mg/dL for hyperlipidemia. Peripheral venous blood samples were collected on admission to the inpatient ward. Complete blood count analysis was performed with an automated blood cell counter (Beckman Coulter analyzer, Brea, CA, USA). The levels of the following blood biochemistry parameters were measured: total cholesterol, triglyceride, HDL-C, LDL-C, and creatinine. 

### 2.3. Statistical Analysis

The results are presented as the mean ± standard deviation (SD) for continuous variables with normal distribution and categorical data as the number and percentage of total patients. *T*-test or one-way ANOVA was used for comparison of continuous variables. A two-sided *p*-value < 0.05 was considered as statistically significant. The correlation of selected variables in CAE patients was estimated by the Pearson test. Logistic regression analysis was used to ascertain independent variables associated with CAE. The results are presented as the odds ratio (OR) with a 95% confidence interval (CI). The statistical analysis was performed with SPSS software (version 21.0). 

## 3. Results

**Figure 1:**
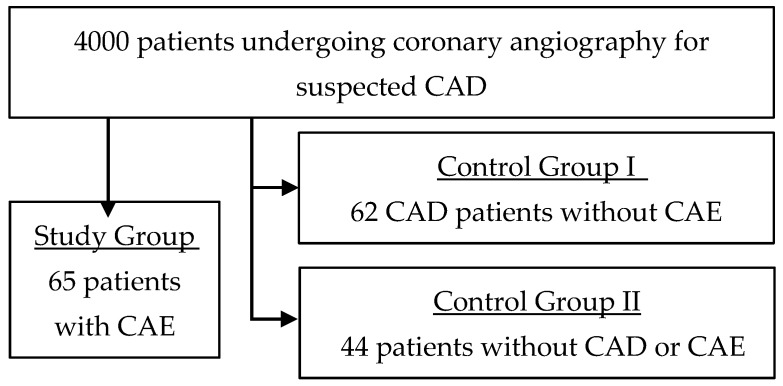
Cohort study flowchart. CAE, coronary artery ectasia. CAD, coronary artery disease.

Over 5 years, the catheterization unit performed 4000 catheterizations, and 171 patients were selected for the study according to the inclusion and exclusion criteria. The study group, or ectatic group, comprised of 65 patients with diagnosed CAE, representing a CAE prevalence of 1.6%. The obstructive group, with no significant CAD, was comprised of 62 patients. The control group included 44 patients with neither significant CAD nor CAE (Figure 1).

Clinical, angiographic, and pharmacological characteristics of the population are presented in Table 1. The height of the patients was significantly different between the groups, especially in the ectatic group (*p* = 0.04). Cardiovascular background diseases were largely different among the groups. The presence of stable angina was mainly observed in the control group (*p* = 0.003). The presence of unstable angina (*p* = 0.001), ST-elevation myocardial infarction (STEMI) (*p* = 0.001), and history of CAD due to myocardial infarction (*p* = 0.02) were significantly higher in the obstructive group. Other comorbidities, including hypertension, diabetes, dyslipidemia, smoking, chest pain, and angina history, were not statistically significant among groups (Table 1). 

Statins and aspirin were prescribed more in the obstructive group (*p* = 0.014 and *p* = 0.001, respectively). The use of diuretics, insulin, angiotensin-converting enzyme inhibitors, angiotensin receptor blocker, calcium channel blockers, nitrates, and oral hypoglycemic drugs was similar among the three groups. A comparison of the biochemical and hematological characteristics indicated a different lipid profile among the groups (Table 2). HDL-C was the highest in the control group, representing patients with normal coronary angiograms (*p* = 0.007), whereas hemoglobin values were higher in the CAE group (*p* = 0.049). LDL-C, total cholesterol, triglycerides, and creatinine levels, as well as echocardiographic measurements, were similar among the population groups (Table 2 and Table 3). 

Echocardiographic analysis indicated reduced ejection fraction (*p* = 0.02) and the presence of abnormal ECG findings (*p* < 0.001) in the CAE group. In the ectatic group, the correlation coefficient analysis demonstrated a negative correlation for HDL-C (r = −0.274, *p* < 0.001) in CAE patients. Logistic regression analysis was performed to find independent predictors for the presence of ectatic arteries. The initial selection of the variables for the univariate analysis with adjusted odds ratios was calculated for height, statin use, aspirin, stable and unstable angina, STEMI, non-ST-elevation myocardial infarction (NSTEMI), history of coronary disease, hemoglobin, ejection fraction, LDL-C, HDL-C, LDL-C/HDL-C ratio, and abnormal ECG. Significant results (*p* < 0.05) were subjected to multivariate regression analysis (Table 4). The multivariate logistic regression analysis showed that HDL-C (*p* = 0.029) and hemoglobin (*p* = 0.021) were independently associated with CAE. In fact, we observed that a one-unit increase in HDL-C led to a 15% risk reduction of CAE. In addition, each unit increase in hemoglobin could potentially increase the CAE risk by 2-fold.

## 4. Discussion

The potential role of lipoproteins in the remodeling process leading to the development of CAE remains elusive. In the present study, we demonstrated, for the first time, an association between plasma lipoproteins and the incidence of CAE in patients with suspected CAD. Overall, CAE patients presented with an intermediate clinical profile between the obstructive and the control group. We found that patients with CAE had significantly lower HDL-C and a higher LDL/HDL ratio compared to the control group with normal coronary angiograms. Our findings are supported in cases of familial hypercholesterolemia [8]. Sudhir and collaborators described an increased prevalence of CAE in patients with familial hypercholesterolemia in the presence of higher LDL-C levels, lower HDL-C, and hence higher LDL/HDL ratio [8]. In a recent study, Qin and collaborators compared cardiovascular risk factors and associations in patients with CAE and patients with CAD [10]. The study indicated that triglycerides and LDL-C/HDL-C ratio also had the predictive potential for CAE when compared to a group with atherosclerosis without CAE. Nevertheless, the prevalence of CAE was not thoroughly investigated in patients with CAD compared to a control group without CAD, as in this study. Additionally, we observed elevated hemoglobin levels as a predictive factor for CAE. We hypothesized that elevated hemoglobin could cause a sluggish blood flow, predisposing subsequent dilation of the artery, potentially leading to increased risk of thrombosis and myocardial infarction [11,12,13]. 

Stable and unstable angina were primarily observed in the control group due to their non-significant CAD component, in turn, the obstructive group was characterized by significant CAD with higher ST-elevation myocardial infarction (STEMI) and non-ST-elevation myocardial infarction (NSTEMI) prevalence rates. Other underlying diseases, such as hypertension, diabetes, smoking, chest pain, and angina history, had a similar frequency among groups. The analysis of clinical characteristics indicated the use of statins and aspirin was more often in the obstructive group as part of the current CAD management. Subsequent multiple regression analysis showed that statins might potentially be a weak indicator for CAE occurrence. We concluded that since most patients with CAD take statins, an altered lipoprotein profile might underlie the pathogenesis of CAE [8,9]. In fact, controversy arises as to whether CAE should be considered as part of the spectrum of atherosclerotic diseases or a separate phenomenon [14,15]. Furthermore, CAE is also observed in other pathologies, including connective tissue diseases (Marfan and Ehlers–Danlos), autoimmune disease (scleroderma, lupus erythematosus, polyarteritis nodosa), bacterial infections (syphilis and Lyme diseases), and in rare cases associated with trauma, congenital origin, cocaine use, or Kawasaki disease [2]. The main limitations of the study are its retrospective character and the relatively small number of patients enrolled due to the low prevalence rate of CAE.

## 5. Conclusions

The results of this study indicate that high HDL-C decreases the risk of developing ectatic arteries, whereas elevated hemoglobin has a strong effect on the development of ectatic arteries. Furthermore, the elevated LDL-C/HDL-C ratio has a predictive value for CAE development. Indeed, our work suggests a link between plasma lipoproteins, hemoglobin, and coronary aneurysms, requiring further study. A larger prospective study must be conducted to monitor the outcomes of these patients and to evaluate the importance of the existing findings.

## Figures and Tables

**Table 1:** Clinical and angiographic characteristics of the study group.

Variable	Ectatic (*n* = 65)	Obstructive (*n* = 62)	Control (*n* = 44)	*p*-Value
**Patient Characteristics**				
Height, cm	175.64 ± 6.62	170.32 ± 9.90	169.61 ± 10.34	0.04
Weight, kg	89.35 ± 12.97	86.14 ± 17.19	81.25 ± 19.24	0.17
BMI	28.96 ± 3.82	29.62 ± 4.92	28.22 ± 6.30	0.49
**Comorbidities**				
Stable angina, *n* (%)	5 (7.7)	2 (3.2)	10 (22.7)	0.003
Silent ischemia, *n* (%)	3 (4.6)	0 (0.0)	2 (4.5)	0.23
Unstable angina, *n* (%)	28 (43.1)	18 (29.0)	29 (65.9)	0.001
NSTEMI, *n* (%)	13 (20.0)	18 (29.0)	4 (9.1)	0.04
STEMI, *n* (%)	22 (33.8)	29 (46.8)	8 (18.2)	0.001
Hypertension, *n* (%)	51 (78.5)	48 (77.4)	29 (69.0)	0.50
Diabetes, *n* (%)	20 (30.8)	18 (29.0)	15 (35.7)	0.76
Dyslipidemia, *n* (%)	54 (83.1)	55 (88.7)	34 (81.0)	0.50
Smoker, *n* (%)	35 (53.8)	37 (59.7)	18 (42.9)	0.23
Prior of angina, *n* (%)	2 (3.1)	4 (6.5)	1 (2.4)	0.51
Prior typical/atypical chest pain, *n* (%)	1 (1.5)	1 (1.6)	2 (4.8)	0.50
Prior CAD, *n* (%)	23 (35.4%)	26 (41.9)	7 (16.7)	0.02
**Pharmacological Treatment**				
Statins, *n* (%)	51 (81.0)	58 (96.7)	27 (79.4)	0.014
Diuretics, *n* (%)	15 (23.8)	15 (25.0)	9 (26.5)	0.95
Insulin, *n* (%)	1 (1.6)	1 (1.7)	2 (5.9)	0.37
ACEi or ARB, *n* (%)	39 (61.9)	45 (75.0)	20 (58.8)	0.18
Aspirin, *n* (%)	55 (87.3)	60 (100)	23 (67.6)	0.001
CCB, *n* (%)	6 (9.5)	12 (20.0)	8 (23.5)	0.13
Nitrates, *n* (%)	7 (11.1)	4 (6.7)	2 (5.9)	0.56
Oral hypoglycaemic, *n* (%)	9 (14.3)	10 (16.7)	9 (26.5)	0.31

Continuous variables are shown as mean ± standard deviation (SD). Nominal variables are presented as a percentage in brackets. BMI, body mass index. NSTEMI, non-ST-elevation myocardial infarction. STEMI, ST-elevation myocardial infarction. CAD, history of coronary artery disease prior to myocardial infarction. ACEi, angiotensin-converting-enzyme inhibitor. ARB, angiotensin receptor blockers. CCB, Calcium channel blocker.

**Table 2:** Biochemical and hematological measurements of the study population.

Variable	Ectatic (*n* = 65)	Obstructive (*n* = 62)	Control (*n* = 44)	*p*-Value
Total cholesterol, mg/dL	185.14 ± 55.44	185.51 ± 54.55	190.39 ± 37.76	0.85
Triglyceride, mg/dL	155.89 ± 87.07	184.26 ± 128.43	159.69 ± 114.34	0.34
HDL-C, mg/dL	40.13 ± 10.15	38.67 ± 10.16	45.52 ± 11.97	0.007
LDL-C, mg/dL	116.61 ± 51.86	110.37 ± 44.03	117.86 ± 32.66	0.67
Total cholesterol/HDL-C ratio	4.76 ± 1.2	4.92 ± 1.11	4.15 ± 0.77	0.001
Triglyceride/HDL-C ratio	3.78 ± 2.24	4.67 ± 2.65	3.43 ± 2.76	0.03
LDL-C/HDL-C ratio	3.02 ± 1.76	2.87 ± 1.53	2.66 ± 1.03	0.001
Hemoglobin, g/dL	14.09 ± 1.76	13.37 ± 1.57	13.49 ± 1.63	0.049
Hematocrit (%)	41.38 ± 5.00	39.33 ± 4.45	39.67 ± 4.35	0.04
Creatinine, mg/dL	1.20 ± 0.80	1.12 ± 0.63	1.23 ± 0.88	0.74

Continuous variables are presented as mean ± standard deviation (SD). HDL-C, high-density lipoprotein cholesterol. LDL-C, low-density lipoprotein cholesterol.

**Table 3:** Echocardiography and ECG characteristics.

Variable		Ectatic (*n* = 65)	Obstructive (*n* = 62)	Control (*n* = 44)	*p*-Value
Rest LV function, *n* (%)	Normal	28 (51.9)	25 (51.0)	27 (75.0)	0.09
	Mild	5 (9.3)	10 (20.4)	3 (8.3)	
	Moderate	10 (18.5)	9 (18.4)	4 (11.1)	
	Severe	11 (20.4)	5 (10.2)	2 (5.6)	
Ejection fraction, mean ± SD		48.55 ± 14.25	50.01 ± 11.28	55.94 ± 11.44	0.02
LV hypertrophy, *n* (%)		20 (38.5)	19 (33.3)	9 (23.7)	0.33
Mitral regurgitation, *n* (%)	Mild	47 (87.0)	58 (93.5)	35 (92.1)	0.45
	Moderate	7 (13.0)	4 (6.5)	3 (7.9)	
Aortic stenosis, *n* (%)	Mild	51 (94.4)	59 (95.2)	36 (97.3)	0.80
	Moderate	3 (5.6)	3 (4.8)	1 (2.7)	
Tricuspid regurgitation, *n* (%)	Mild	51 (96.2)	59 (95.2)	37 (97.4)	0.85
	Moderate	2 (3.8)	3 (4.8)	1 (2.6)	
Pulmonary pressure (mmHg), mean ± SD		25.48 ± 6.54	25.86 ± 11.07	23.96 ± 7.08	0.63
ECG pathological findings, *n* (%)		21 (35.6)	4 (7.4)	5 (11.9)	<0.001

Continuous variables are presented as mean ± standard deviation (SD) or *n* (%). Nominal variables are presented as a percentage in brackets. LV, left ventricular. ECG, echocardiography.

**Table 4:** Multiple logistic regression analysis showing independent predictors of CAE.

Variable	*p*-Value	OR	95% CI	
			Lower	Upper
LDL-C/HDL-C ratio	0.034	1.987	1.542	2.882
HDL-C, mg/dL	0.029	0.858	0.749	0.984
Hemoglobin, g/dL	0.021	2.060	1.114	3.809
Statin use	0.060	16.562	0.890	308.351

CI, confidence interval. OR, odds ratio. CAE, coronary artery ectasia. Only the influencing variables were introduced.
